# Development of an intervention program to increase effective behaviours by patients and clinicians in psychiatric services: Intervention Mapping study

**DOI:** 10.1186/1472-6963-10-293

**Published:** 2010-10-25

**Authors:** Bauke Koekkoek, Berno van Meijel, Aart Schene, Giel Hutschemaekers

**Affiliations:** 1 ProPersona Mental Health Care, Pro Persona Centre for Education and Science, Wolfheze; 2 Altrecht Mental Health Care, Zeist, The Netherlands; 3 InHolland University for Applied Sciences, Research Group Mental Health Nursing, Amsterdam, The Netherlands; 4 Department of Psychiatry, Academic Medical Centre, University of Amsterdam, Amsterdam, The Netherlands; 5 Radboud University, Academic Centre of Social Sciences, Nijmegen, The Netherlands

## Abstract

**Background:**

Health clinicians perceive certain patients as 'difficult' across all settings, including mental health care. In this area, patients with non-psychotic disorders that become long-term care users may be perceived as obstructing their own recovery or seeking secondary gain. This negative perception of patients results in ineffective responses and low-quality care by health clinicians. Using the concept of illness behaviour, this paper describes the development, implementation, and planned evaluation of a structured intervention aimed at prevention and management of ineffective behaviours by long-term non-psychotic patients and their treating clinicians.

**Methods:**

The principles of Intervention Mapping were applied to guide the development, implementation, and planned evaluation of the intervention. Qualitative (individual and group interviews), quantitative (survey), and mixed methods (Delphi-procedure) research was used to gain a broad perspective of the problem. Empirical findings, theoretical models, and existing evidence were combined to construct a program tailored to the needs of the target groups.

**Results:**

A structured program to increase effective illness behaviour in long-term non-psychotic patients and effective professional behaviour in their treating clinicians was developed, consisting of three subsequent stages and four substantial components, that is described in detail. Implementation took place and evaluation of the intervention is being carried out.

**Conclusions:**

Intervention Mapping proved to be a suitable method to develop a structured intervention for a multi-faceted problem in mental health care.

## Background

In various health care settings, clinicians perceive particular patients as 'difficult'. 'Difficult' is an individual judgment that generally refers to patients who have limited social functioning, make high use of medical services, and generally are unsatisfied with the care they receive [1-6]. The more of these elements are combined and the smaller the perspective of future recovery, the more likely it becomes that a patient is perceived as 'difficult' by a professional. In psychiatric services, most 'difficult' patients are found among patients with long-term non-psychotic illness as mood, anxiety, substance use, and personality disorders that have not responded well to previous treatments [7]. Patients perceived as 'difficult' may be labelled as such in services, and subsequently be at increased risk to be treated less respectfully, less effectively, and to be excluded from health services because of their failure to comply with its implicit and explicit rules for 'proper' patienthood [8-10]. Professionals working with these patients report more stress and burn-out [11,12].

It is not unusual for mental health professionals to ascribe problems in treatment to patients through the use of the 'difficult'-label. This routine has been criticized repeatedly [13-15]. Indeed, it is not always clear which patient behaviours must be considered as originating in the psychiatric disorder itself, and which may be the consequence of an ineffective contact with mental health clinicians or services [16]. In previous work, for instance, we found no association between any specific non-psychotic psychiatric disorder and clinicians' 'difficult'-judgment [17]. However, clinicians' perceptions of the patient (e.g. seeing the patient as able but unwilling to change), the patient's previous service use and the number of psychosocial problems, were independently associated with clinician-perceived difficulty [17]. Thus, patients' responses to illness and treatment (*illness behaviour*) may prevail over the illness itself. Since the concept of illness behaviour not only refers to the different ways in which people perceive, evaluate, and respond to symptoms [18], but also to the ways in which they seek help and to their behaviour in healthcare systems, this concept is highly relevant to the understanding and prevention of perceived difficulty. Recurring behaviours that are perceived as difficult by clinicians may be described as 'ineffective chronic illness behaviour', which in part may result in 'ineffective professional behaviour' as a response [19]. We therefore use these terms to describe certain 'difficult' behaviours by long-term patients with non-psychotic disorders (e.g. constant complaining about ever-changing problems, recurrent making of suicidal threats, repeated denial of financial problems) and certain ineffective responses by professionals (e.g. not listening to patients' long-term problems, responding only to acute problems, failure to intervene in obvious social problem situations).

Currently, prevention of these two types of ineffective behaviour is not a high priority in mental health services. In general, the management of non-psychotic chronic patients in psychiatric care is poorly developed. While evidence-based treatments for various non-psychotic disorders are available, they are not for non-psychotic *chronic *disorders. Although some treatments exist for specific subgroups (e.g. chronic depression [20,21] and borderline personality disorder [22,23]), they do not apply to the entire target population [24,25], of which some patients may not (yet) be ready for such treatments. The project described in this paper aims at the development of an intervention program to both prevent and manage these ineffective behaviours among long-term non-psychotic patients who have not benefited from previous treatment, and their key clinicians.

## Methods

Intervention mapping (IM), a systematic method for the development, implementation and evaluation of health interventions outlined by Bartolomew et al. [26,27], has proven to be a useful way to construct programs grounded both in theory and empirical data [28,29]. IM proceeds according to the following steps. Step 1 consists of a needs assessment through a review of the scientific literature to analyse the target population, environmental conditions, and determinants of health behaviour. In step 2 the determinants of the health behaviour are used to set objectives for behaviour change, divided in broad performance objectives and concrete change objectives in terms of what a person needs to learn to change his or her behaviour. In step 3, theoretical foundations and empirically evaluated methods and strategies for behaviour change are assessed. In step 4, the methods and strategies are translated into an organized intervention. In step 5, the adoption, implementation and sustainability of the intervention is planned. In step 6, an evaluation plan is provided for and carried out. The strategies used in this project for each of the six steps in Intervention Mapping are reported on in detail below.

For step 1 and 2, we carried out a comprehensive review of the literature on 'difficult' patients. The MEDLINE, CINAHL, and PsycINFO databases were searched for English articles published between 1979 and 2004, retrieving 94 eligible papers [7]. Next we undertook additional research to describe the health behaviour and its determinants: a qualitative interview study among patients [30], a survey among community mental health clinicians [17], and a Delphi-exercise among scientists/policy makers/expert-professionals [16,19,25,31]. We concluded with the formulation of the overall behavioural objective of the intervention, and the more concrete change objectives.

For step 3, we made a theoretical analysis of ineffective chronic illness behaviour [Koekkoek B, Hutschemaekers G, van Meijel B, Schene A: How do patients become to be seen as 'difficult'?: a mixed-methods study in community mental health care, revision submitted], which forms the foundation of the intervention program. We conducted a review of therapeutic methods available to change determinants (assessed in step 1), reach objectives (formulated in step 2), and confront ineffective behaviours of both patients and professionals [search strategy and results available from the 1^st ^author]. Additionally, since empirical findings were limited, we collected data from current best practice sites. We visited three well-known national best practices, specialized in three important domains of long-term non-psychotic disorders (mood disorders, substance abuse disorders, and personality disorders) for data on possible effective practice-based strategies not yet described in the literature. Selection of these best practices took place by searching national scientific and professional journals, searching conference programs and reports, and inviting leaders in the fields (e.g. professors, directors, educators) to suggest best practices.

For step 4, we consulted an expert group of clinicians, scientists, and policy makers over an extended period of time (two years). Some of these experts were participants in one of the problem analysis studies in step 1, others were invited because of their expertise in a specific therapeutic method (for instance clinical case management or behaviour therapy).

In step 5, implementation was prepared with a steering group of scientists and managers in the psychiatric service the intervention was tested in. Before an agreement was reached, the intervention was first presented to a director, a research psychiatrist, and the psychiatrist of the team in which the intervention would be implemented. Next, the intervention and its evaluation were presented to the team members who all agreed to participate. After obtaining ethical permission and the final approval of the institution's chief director, the program was implemented.

In step 6, we designed a mixed-methods pilot study to evaluate the intervention program. This pilot study consists of quantitative and qualitative measurements of outcome and process variables, and is described in more detail later.

Ethical approval was obtained for the patient-related qualitative study and the pilot study from the Institutional Review Board of the organisation the 1^st ^author is affiliated with. Informed consent was obtained from all participating patients in aforementioned patient-related studies.

## Results

Outcomes of the Intervention Mapping process will be described according to the six steps.

### Step 1: Needs assessment

#### Analysis of target population

Non-psychotic psychiatric disorders are highly frequent in the general population: lifetime prevalence in the US is 28.8% for anxiety disorders, 19.1% for depressive disorders, 14.6% for substance use disorders [32] and 9.1% for personality disorders [33]. Comparable percentages were found in The Netherlands, United Kingdom, Australia, and other Western countries [34-36]. Together these disorders account for the majority of mental illness in the community, and some of these become chronic. The percentage of non-psychotic patients in long-term care is estimated between 20 and 50% [37-41]. Of these patients, about 28% is perceived as difficult by psychiatric clinicians [17].

#### Analysis of environmental conditions

Even though the prevalence of non-psychotic psychiatric disorders in The Netherlands, where this study took place, is comparable to that of other countries, the availability of services may be somewhat different. The Dutch mental health care system is paid for by a mixture of federal grants, individual health insurance, co-payment and fee-for-service. However, long-term care for patients with non-psychotic disorders is fully paid for by federal budgets, even for those without insurance. Compared to the USA, Canada and Puerto Rico, a substantially higher percentage of people is treated in mental health services, both in general and specialty health care [42]. Also financially, there are few limits on the availability of long-term care in The Netherlands, compared to other countries [43].

#### Analysis of behaviour

We found three types of behaviours to be specific to perceived difficulty in mental health care. First, the presence of various psychiatric symptoms that are inconsistent, shifting, temporal and thus prohibiting the making of a clear diagnosis for which treatment can be started. Second, the presence of unusual help-seeking behaviour and interpersonal behaviour that is for instance chaotic (actively seeking help for constantly shifting problems with various agencies), dependent (actively seeking continuation and intensification of help), or ambivalent (actively seeking but not accepting help) that is poorly understood by psychiatric professionals. Third, the presence of various social problems (e.g. debts, poverty, poor housing, unemployment, difficulties in upbringing of children, legal issues etc.) that patients appear to consider as mental health problems but that can often not be solved by psychiatric professionals.

Some of these problems (for instance the described forms of unusual help-seeking) may be typical for people with non-psychotic disorders, others may also apply to people with psychotic disorders. In psychiatric services, however, mental health professionals still seem to hold different views on non-psychotic disorders (generally seen as transient, psychological problems) and psychotic disorders (generally seen as chronic, neurobiological problems) [7]. As such, professionals tend to consider long-term non-psychotic patients largely as responsible for their problems. Subsequently, professionals are ambivalent about considering these patients as chronically ill, and about reinforcement of their claim to the sick role. This ambivalence about legitimateness of chronic illness may cause friction in the therapeutic relationship, resulting in the qualification of the non-psychotic patient as a 'difficult' patient.

#### Analysis of behavioural determinants

From our literature review and subsequent research studies we concluded that other than patient-related factors are equally relevant in the occurrence of difficulties in the care of non-psychotic chronic patients [7]. While patient-related factors solely focus on, for instance, psychopathology, there are more variables that account for difficulties. Such variables could be categorized in four groups. The 1^st ^is professional-related (e.g. the professional's willingness to engage with long-term patients). The 2^nd ^is interaction-related, (e.g. the quality of the contact between patient and professional). The 3^rd ^is social system-related (e.g. the amount of social support a patient has outside the mental health care system). The 4^th ^is mental health care-related (e.g. the support a professional receives from co-workers and managers to care for long-term patients).

The mental health care-related category was by far the most relevant according to experts. General professionals laid more emphasis on social factors and less on specific diagnoses, professional skills or mental health care factors. Patients, in turn, stressed the importance of the professional's competencies, the quality of the patient-professional interaction and the views on non-psychotic chronic patients held in psychiatric services. As such, ineffective chronic illness behaviour appears to be the consequence of a complex interplay of factors, while these factors are viewed differently by distinct interest groups. Table 1 shows the determinants from our aggregated results, distinguished by patients, professionals and experts.

**Table 1:** Determinants of ineffective chronic illness behaviour according to research findings among three interest groups

Interest group	Determinant
**Patients**	- Lack of empathy in professional - professional pessimism

**Professionals**	- Lack of social support - professional pessimism

**Scientists/policy makers/expert-professionals**	- Unusual help-seeking style of patients - Lack of professional skills - Lack of view on problems - Lack of suitable and structured treatment - Lack of organisational support

### Step 2: Matrix of change objectives

Based on the needs assessment, the overall behavioural outcome was defined a*s 'an increase of effective behaviours in people with long-term non-psychotic mental illness and their treating professionals*'. We established that current ineffective behaviours consist of ineffective chronic illness behaviour by patients, and ineffective professional responses or behaviour by clinicians. These behaviours are caused by several patient-related and non patient-related determinants, and therefore performance objectives should be set on the patient, professional and services level. Next, important and changeable determinants of behaviour need to be chosen. For each of the three (patients, professionals and services), one determinant, taken from table 1, is exemplified in more detail (table 2).

**Table 2:** Matrix of intervention objectives for each target group

Target group	Determinant (selected)	Performance objectives	Change objectives
**Patient *&*Social system**	Unusual help-seeking style	- Patient decides to negotiate expectations with clinician - Patient and professional reach or maintain a positive working alliance	- Decides to accept increased autonomy offered by mental health care professionals - Uses this autonomy to discuss treatment form and content with professional

**Professional**	Professional pessimism	- Professional expresses a neutral view on behaviour, disorder and treatment results of his/her patients - Professional and patient reach or maintain a positive working alliance	- Decides to consider own view of patient's behaviour, disorder and treatment results as partly responsible for ineffective chronic illness behaviour. - Decides to follow training and supervision on how to look at patient behaviour more neutrally. - Actively participates in supervision meetings on this subject. Supports colleagues in using such skills

**Psychiatric service/Psychiatric profession**	Lack of view on problems	- Service or treatment team expresses a coherent view on the treatment of non-psychotic chronic patients	- Develops and endorses a view of chronicity of non-psychotic patients as partly caused by mental health care itself - Offers training and supervision to increase professionals' skills and attitudes - Enables regular evaluative meetings of skills of professionals and effects on patients - Enables supervision meetings for professionals to offer mutual support and further development of a mutually shared view

### Step 3: Theoretical methods and practical strategies

Far most theoretical models of illness behaviour focus on individuals' help-seeking behaviour and decision-making process before entering the health care system [44]. Few specifically consider illness behaviour of people with psychiatric problems, which appears to differ qualitatively from illness behaviour related to physical problems [44]. A notable exception to this observation is the Network Episode Model [45], that combines the perspective of an illness career with social, cultural, medical and economical variables into a dynamic perspective. Developed by social scientists, this model however is still too general to explain the occurrence of ineffective illness behaviour within psychiatric services. We have, therefore, developed a more detailed model to describe the occurrence of ineffective chronic illness behaviour [Koekkoek B, Hutschemaekers G, van Meijel B, Schene A: How do patients become to be seen as 'difficult'?: a mixed-methods study in community mental health care, revision submitted]. The model shows that the 'difficult'-patient label is given by professionals when certain patient characteristics are present and a specific causal attribution about the patient's behaviours is made. The status of 'difficult' patient is easily reinforced by subsequent patient and/or professional behaviour, turning initial unusual help-seeking behaviour into 'difficult' or ineffective chronic illness behaviour. Furthermore, a lack of resources in the psychiatric service and the patient's social system negatively influence the patient-professional interaction.

The tentative model differentiates between five stages of the treatment process. In stage 1, patient characteristics guide the professional's appraisal process, who labels the patient either or not 'difficult' based on the attribution of patient behaviour (stage 2). As stated earlier, professionals have few resources available on the treatment of these long-term non-psychotic patients [46-48] and therefore are easily demoralized about treatment effectiveness. At the same time, both patients and the general public may have high expectations about cure for these patients, who are sometimes referred to as the 'worried well' [49]. Not only does this term underestimate patients' difficulties, it also pays little attention to the conflicting demands (few resources, high expectations) laid upon clinicians. Clinicians tend to respond with limited involvement and pessimism, which may result in undertreatment (stage 3) or blaming the patient for being ill or not getting better. In stage 4, professional responses to the now-labelled 'difficult'- patient may make the patient conclude that the professional is uncaring or unwilling to offer help. Thus, the patient, with many complex problems and a different style of help-seeking, is confronted with a negative and pessimistic attitude of the professional, resulting in a low dosage of help that aims for management, not recovery. This low-dose help reinforces the original behaviour of patients in distinct ways, thus leading to repetition, perpetuation and even aggravation of the initial problems. In stage 5, patient and professional are reinforcing each others ineffective behaviours based on their previous attributions. These behaviours may have little to do with the problems the patient initially sought help for. In fact, patient and clinician enter a vicious cycle of ineffective chronic illness behaviour (patient) and ineffective chronic professional behaviour (clinician) [Koekkoek B, Hutschemaekers G, van Meijel B, Schene A: How do patients become to be seen as 'difficult'?: a mixed-methods study in community mental health care, revision submitted]. From this theoretical model we have conceptualized the following stages in the intervention program (table 3) - each fitting an important step in the theoretical model.

**Table 3:** staged intervention program based on theoretical model and empirically validated methods

Treatment Stage	Stage I		Stage II	Stage III
**Goal in intervention program**	- Alternative understanding of patient's behaviour - Optimization of working alliance		Clarification of and agreement over goals and tasks	Improvement of psychiatric and social functioning

**Understanding from theoretical model**	- Non-blaming attribution of behaviour by clinician increases chances of positive working alliance - Mutual clarification of expectations increases chances of mutually supported conceptualization of sick role		Active and mutual goal-setting by clinician and patient improve chances of patient's positive attribution and restoration of professional's belief in treatment	Practical and real help improves chances of patient's effective illness behaviour and professionals' effective behaviour

**Empirically validated method**	Team supervision & monitoring through feedback and report forms	Relationship management & motivational interviewing	Motivational interviewing & shared-decision making	Clinical case management & behavioural analysis

### Step 4: Intervention

In this stage, the theoretical model (described above) and practical methods (described in detail in section 3 of this step) were translated into a manual for the intervention, which we named Interpersonal Community Psychiatric Treatment (ICPT) since the interpersonal contact between patient and professional is the main target of the intervention.

The intervention is designed for use in departments or programs for long-term ambulatory care, to which patients may be referred when short-term treatment, aimed at cure, has been found unsuitable or unsuccessful. In such departments, long-term care tends to turn into an unstructured, aimless, and sheer endless enterprise. Professionals working within these department are used to working with long-term patients with a severe mental illness. Often they do this autonomously but share the clinical responsibility with a doctor or psychiatrist, who has the final medical responsibility and sees the patient at a low frequency.

From our descriptive studies and the theoretical model we concluded that an intervention program should focus on: (1) a clear generic treatment structure (to prevent uninformed and haphazard low-dosage help), (2) a phased model (which fits the patient's level of acceptance of help), (3) a therapeutic style that fits the phase the patient is in, (4) a routine monitoring of the interpersonal contact between patient and (5) professional, and support for team professionals.

#### (1) Generic structure

Based on various evidence-based treatments of specific non-psychotic disorders [20,22], we introduced a fixed structure for each session, taking 45 minutes as the standard duration. The first 5 minutes are used by the clinician and the patient to set a mutually agreed on agenda for the session, including themes and goals to be discussed. The next 5 minutes are used to look back from the current to the previous session, allowing a process-oriented discussion of the patient's current mental state and that of the elapsed time since the last session. In the following 25-30 minutes the themes, subjects and goals that have been set on the agenda, are discussed and summarized. The last 5 minutes are used to look back on the session and to fill out a report form (clinician) and a feedback form (patient), which will be exemplified below.

#### (2) Stage model

This model is an explication of the three stages described above (table 3), moving from the 1^st ^stage (optimization of working alliance), through the 2^nd ^stage (clarification of and agreement over goals and tasks) to the 3^rd ^stage (improvement of psychiatric and social functioning). In order to optimize the patient-professional interaction across all stages, it is crucial for the clinician to determine in which stage the treatment contact is located. Clinicians may ask themselves 'diagnostic' questions related to each stage. There are two or three such questions per stage, which are thought and asked during the training and supervision sessions. For the 1^st ^stage such a question is for instance 'do I feel the liberty to discuss the nature of the treatment contact with my patient?'. If the answer to this question is 'no', for example because the professional fears that the patient will become very anxious to lose the treatment contact, the clinician knows that the contact still is in the first stage of optimization of the alliance. As such, the change objective of 'being able to discuss form and content of treatment' (table 2) may not have been reached yet. The stage model helps professionals to structure their treatment, using different methods across different stages.

#### (3) Therapeutic methods per stage

One of the crucial elements of ICPT, in order to prevent ineffective illness and professional behaviour, is the differentiation of therapeutic styles across treatment stages. This approach is a variation of, but consistent with, the trans-theoretical model of change [50] which differentiates people's readiness to change into various stages. In the 1^st ^stage, in which the working alliance is defined, the suggested methods are *relationship management *[51-53] and *motivational interviewing *[54,55], of which especially the latter has a firmly established empirical base. Both methods aim to prevent the usual mental health care 'script' in which the clinician is the one who looks for problems in the patient, and suggests improvements of his or her behaviour, while the patient is a passive recipient of help. Instead, in both methods the clinician is a careful and observant listener who elicits timely responses from the patient and strongly promotes autonomy. In relationship management, the basic rule is to do no harm - referring to the adverse outcomes that have been reported with patients that do not respond well to an actively helping clinician [23,53,56]. Motivational interviewing seeks to create and increase patient's ambivalence, for instance by juxtaposing riskfull behaviour with responsible parenthood in a person who loves his or her child but also engages in repeated self-destructive behaviours.

In the second stage of ICPT, motivational interviewing is used again in a generic way, now to set patient-centred goals. It is complemented with *shared decision making *[57]. This method, imported from physical health care, makes use of a structured way to make treatment decisions mutually agreed on by patient and professional. We added systematic *goal-setting *to this procedure. After an initial open question to focus the patient on the future ('what do you want your life to look like in one year from now?'), a more detailed analysis follows of the areas where change is desired. Then, aided by a widely used tool to assess care needs [58] which identifies possible unmet needs that may obstruct progress, specific goals are jointly formulated. This careful process of mutual goal setting seeks to avoid common pitfalls: the patient feeling that treatment goals are forced upon him or her, and the clinician feeling that urgent patient needs (e.g. financial problems) have not come under discussion.

In the third stage of ICPT, two different goal-oriented methods are used to improve personal and social functioning. The more practical variant, often required with patients that have many social problems, is *clinical case management *[59-61]. This form of psychiatric case-management assumes one responsible clinician who takes an active role to improve the patient's social situation, through helping solving social problems (e.g. problems with housing, income, debts, social activities etc.). This form of case-management is, despite its lower implementation grade than the earlier mentioned Assertive Community Treatment [59], more suitable to situations in which team-wise treatment is not possible. The second variant, possible with patients who have less severe social problems, is *behavioural analysis*. This generic and empirically supported form of focused behaviour therapy [20,62], assumes that people with long-term non-psychotic disorders mostly find themselves caught in unsatisfactory interpersonal situations. These situations become object of analysis in a stepwise behavioural protocol [20] which focuses on the thoughts, feelings, actions and consequences regarding the patient's interpersonal behaviour. This third stage of ICPT, that may not be reached by all patients, aims to offer true, practical help after goal-setting in stage two has been concluded.

#### (4) Application of feedback forms

Originally intended for research purposes, feedback forms have gained solid ground in mental health care over the last years. In ICPT, both clinician and patient fill out a form about the session they have just had. Both rate items on the Session Rating Scale [63], thereby informing one another on their (dis)content with the working alliance. In addition, clinicians score in which stage of the treatment contact this session could be located, as well as which methods were used, and if treatment goals were discussed. Patients, on the other hand, rate their own input in the session's content. By these means, both parties are delegated responsibility for the working alliance and their substantive input in the session.

#### (5) Supervision

Every two weeks, a team-wise supervision takes place in which a treatment situation of two different clinicians is jointly analysed. The stage model is implicitly used by the supervisor, but not forced upon the participants. After a 3-minute description, or through a previously distributed paper sheet with 7 preset questions, the treatment situation is introduced by one of the clinicians. After a 25-minute discussion, the process is finalized by the clinician who introduced the situation, through a short summary and mentioning of learning points. Supervision has been proven to be helpful to reduce stress in community psychiatric nurses [64,65]. We used a brief version of a supervision protocol that has been developed and evaluated in Dutch long-term mental health care [66]. It focuses on the professionals' feelings that may be evoked by working with patients who seem to miss the capacity to improve their independent functioning, are not able to solve their often broad set of psychosocial problems, and have a high level of demands of which they expect the professional to take responsibility for.

### Step 5: implementation

A community mental health team consisting of six community psychiatric nurses and two psychiatrists, with a case-load of severely mentally ill patients with both psychotic and non-psychotic disorders was selected as suitable for a pilot study of the intervention. This selection was based on three criteria: (1) representativeness of the psychiatric service and its catchment area, (2) preparedness and possibility of implementing a new treatment program in the service, (3) geographical accessibility of the service for the authors. Implementation was supported by the management team early on, the clinical team was invited to two meetings about the content and form of the program before the final consent for implementation was given. The team also expressed their willingness to participate in group supervision sessions during the research period. Although this may not be the case in other teams, many professionals express their wish to participate in supervision in daily practice. The team-leader, one of the participating clinicians with additional management tasks, and the team psychiatrist functioned as the link between the treatment team and the research team.

The intervention was implemented mainly through a 3-day training program, consisting of the following elements: (1) theoretical overview (4 hours), (2) relationship management skills (8 hours), (3) motivational interviewing and goal setting skills (4 hours), (4) case-management skills (4 hours), and (5) behavioural analysis skills (4 hours). The training was offered by the first author (8 hours), and four specialists in the specific skills (4 hours each). It combined lectures, group discussions, one-on-one and group-wise role-playing, homework assignments, and self-study of provided literature. Substantial effort was put in tailoring the training program to the needs and competencies of the participants. Many of the existing therapeutic approaches for patients with non-psychotic disorders, are aimed at Master-level clinicians, whereas the participating community psychiatric nurses, the key clinicians of patients and also those intended to carry out ICPT, all had Bachelor-level psychiatric nursing qualifications. Tailoring was done by inviting specialists with extensive experience with both the target group of professionals, and the method to be taught.

Report and feedback forms were fully integrated into the institution's electronic patient file, to facilitate easy use of these forms and the intervention program in general. The training program was followed up by biweekly supervision sessions and hands-on support by email, telephone or face-to-face contact, delivered by the first author. Every two weeks, a 30-minute group-wise booster session took place, designated for the answering of questions about, and enhancement of adherence to ICPT.

### Step 6: evaluation

Scientific evaluation of the intervention is part of the implementation process. For various reasons a pilot study was designed to investigate the feasibility of the intervention. First, little experience has been developed so far with the implementation of community psychiatric nurse-led interventions. We need to consider that the application of ICPT places high demands on professionals' skills. Therefore, biweekly supervision and constantly available coaching by phone, email or live instruction were offered. It is possible though that some of the interventions may not be successfully carried out by nurses. Although we believe, based on prior experience and preliminary results from the pilot study, that nurses are able to do so, a thorough process evaluation is included. Second, likewise, implementation of innovative programs for the target group of patients with long-term non-psychotic disorders has been scarce. Third, the intervention consists of multiple components of which the individual effectiveness is established, yet not in conjunction with other methods. It may be that the application of several treatment strategies within one integral program weakens the effect of the individual interventions - especially when less thoroughly implemented (e.g. through fewer training hours) than in the original research studies. Fourth, this implementation will be used to improve the intervention and to assess the applicability of several patient-administered measures with this patient group, since they are used only with other groups of patients (e.g. patients with psychotic disorders, short-term patients). Positive results of the pilot study may well result in the design and execution of a randomized controlled trial.

This pilot study will have a duration of six months and both quantitative and qualitative assessments will be made at baseline, 3 months and 6 months. Quantitative assessments will include outcome measures (psychopathology, psychosocial functioning, quality of life) and process measures (service use, treatment satisfaction, and quality of the therapeutic alliance) on patient level. It will also include process measures on the professional level (treatment integrity, work satisfaction, and perceived difficulty). Qualitative interviews will be used to assess the feasibility and usefulness of the intervention program among patients and professionals alike. Among clinicians, satisfaction with the training, the program, the support, and the supervision will be investigated quantitatively (through scores) and qualitatively (through interviews).

## Discussion

In this paper we described the systematic development of an intervention program aimed at people with long-term non-psychotic disorders, Interpersonal Community Psychiatric Treatment (ICPT), carried out by community psychiatric nurses in order to prevent ineffective illness and ineffective professional behaviour. By following the steps of the Intervention Mapping process, it has become increasingly clear that behaviours by health clinicians and (illness) behaviours by patients are mutually reinforcing. Thus, this intervention aims not only to change patient's behaviours, but also to change clinicians' behaviours. In fact, patient's behaviours should change through different clinicians' behaviours. A three-stage treatment model was developed, with tailored therapeutic interventions applied in each stage. Implementation mainly took place through a training program, evaluation through a pilot study.

Although the stage model and therapeutic modalities used in this intervention program are relatively straightforward, the health problem it targets is quite complex, and may be more precisely described as an interaction problem within health services. More than in descriptions of other programs aimed at prevention of ineffective health behaviour [e.g. [27,28]], the patient behaviour in this area is very much influenced by the behaviour of health clinicians, and the organisational arrangements of the health services. Ineffective chronic illness behaviour can certainly not be ascribed to patients alone, and therefore an intervention program should also target other parties involved. Although it may appear unusual to target health clinicians' behaviours and health services' policies through an intervention program, in fact many patient behaviours are quite strongly associated with clinicians' professional behaviour [e.g. [67,68]]. Even though studies into the primary or secondary prevention of ineffective chronic illness behaviour are relatively scarce [e.g. [69-71]], we believe that in many health settings, mechanism of mutual reinforcement of ineffective behaviours are relevant but poorly recognized and understudied phenomena. However, the consequences of such reinforcement may be stronger in our population of non-psychotic patients in long-term mental health care. For both patients and professionals, several disincentives (e.g. motivational, financial, and social) may be present in long-term mental health care, requiring a program explicitly aimed at prevention of ineffective behaviours.

The program combines effective methods on various levels. First, it is grounded in the principles of systematic care planning. The stage model of treatment and the generic session structure offers an overall systematic framework. Next, effective therapeutic methods fill this framework with content. Another level encompasses structured feedback professionals receive from their patients, which facilitates improvement of care. At another level, patient-professional cooperation and patient empowerment are important principles that place patient's autonomy in the middle ground. Last, mutual professional support through supervision is an essential element to improve quality and inter-professional cooperation. Several elements of ICPT can be found elsewhere in more detail (for instance goal-setting is very well defined in psychiatric rehabilitation [72]). To our knowledge, however, it is the first time that a number of potentially effective methods is combined into one, ready-to-use program tailored to this patient and professional population - which both have been deprived of theoretical and methodological developments for long.

This study has limitations and strengths. First, large-scale research into the determinants of ineffective chronic illness behaviour is absent. Therefore we had to rely on smaller, though well-focussed, studies. Second, the scope of our findings may be limited by the specifics of the Dutch health care system. As has been noted before, long-term mental health care is relatively well reimbursed in the Netherlands, which may not be so in other countries. However, this limitation applies less to European countries than to the United States, since many European nations have some form of public care for severely mentally ill patients. Third, some elements that are considered important by some, are not present in ICPT. For instance, the patient's social functioning is primarily supported indirectly, i.e. through active encouragement and practical help by the professional, yet not through direct involvement of patient's significant others. Although certainly not discouraged, the introduction of significant others into the mental health care contact is not the program's main goal, which is the optimization of the patient-clinician contact first. Generalization of this improved interpersonal skills is aimed for, however, through the use of behavioural analysis of interpersonal problems. Whether this strategy is sufficiently helpful to improve the patient's social support is to be determined through the pilot study. Fourth, the therapeutic methods chosen for inclusion in ICPT may not be the only ones possible but we have given preference to those methods that best matched the behavioural determinants *and *had most empirical support. Fifth, whether the key clinicians delivering ICPT, community psychiatric nurses, are able to do so effectively after three days of training in a variety of concepts and methods, needs to be assessed empirically. While the intervention program is full, and the training rather short, follow-up is intensive through constant support and biweekly supervision sessions. Sixth, this intervention program might also have been developed using other methods to derive at health care interventions, for instance the MRC Framework [73]. In this paper, we have not reviewed this and other methods in detail since at an earlier stage we found that Intervention Mapping's strong emphasis on intervention development in general, and goal setting and explication of target groups in particular, suited the complex background of the health problem well. We do acknowledge, though, that other models might have been equally applicable.

One of the strengths of this study is the investigation of the health problem from a variety of angles. Also, the patient's perspective has been researched in substantial detail. Furthermore, the theoretical model has been developed over a period of four years and has been exposed to various rounds of feedback from researchers, practitioners, and patients. These measures, to our belief, have greatly increased the validity of our findings.

## Conclusions

Systematic development of an intervention program for a complex health behaviour problem is possible with Intervention Mapping although the method places high demands on clarification of targeted behaviours, determinants, and target groups.

## List of abbreviations

IM: Intervention Mapping; ICPT: Interpersonal Community Psychiatric Treatment

## Conflict of interests

The authors declare that they have no competing interests.

## Authors' contributions

BK, BvM, AS and GH devised the idea of the study and designed the methods. BK led the data collection, analysis, and prepared the manuscript. BvM, AK, AS and GH co-drafted the manuscript. All authors read and approved the final manuscript.

## Pre-publication history

The pre-publication history for this paper can be accessed here:

http://www.biomedcentral.com/1472-6963/10/293/prepub
